# Vascular Endothelial Growth Factor Single Nucleotide Polymorphism +405 G/C (rs2010963) is associated with Levels, Infection Severity, and Amputation among South Indian Diabetic Foot Ulcer Patients

**DOI:** 10.1155/2023/2059426

**Published:** 2023-04-13

**Authors:** Priyanka Ganapathy, Vidya Devanatha Desikan Sheshadri, Rajesh Sarkar, Sumathi Jones, Krishnamoorthy Gunasekaran, Teka Obsa Feyisa, Dhamodharan Umapathy, Saleem Basha

**Affiliations:** ^1^Department of Physiology, Sree Balaji Medical College and Hospital, Chennai, Tamilnadu, India; ^2^Department of Pharmacology and Toxicology, College of Pharmacy (Women's Campus), Prince Sattam Bin Abdul Aziz University, Al-Kharj, Saudi Arabia; ^3^Department of Medical Microbiology, College of Health and Medical Sciences, Haramaya University, Dire Dawa, Ethiopia; ^4^Department of Pharmacology and Therapeutics, Sree Balaji Dental College and Hospital, Chennai, Tamilnadu, India; ^5^Department of Medical Biochemistry, College of Medical and Health Sciences, Dambi Dollo University, Oromia Region, Ethiopia; ^6^Department of Medical Biochemistry, College of Health and Medical Sciences, Haramaya University, Dire Dawa, Ethiopia; ^7^Department of Biotechnology, D.K.M. College for Women, Vellore, Tamil Nadu, India; ^8^Department of Research, APRAISE, Adhiparasakthi Dental College and Hospital, Melmaruvathur, Tamilnadu, India

## Abstract

**Background:**

The regulation of vascular endothelial growth factor (VEGF) by genetic factors in T2DM and DFU still requires thorough investigation. Hence, the present study aimed to investigate the association of VEGF +405 G/C in DFU subjects and correlate it with its circulatory levels, infection severity, and amputation rate.

**Materials and Methods:**

This study registered a total of 754 participants of which group I: healthy controls (*n* = 297), group II: T2DM subjects (*n* = 242), and group III: DFU subjects (*n* = 215). Genotyping and levels of rs2010963 were analyzed by polymerase chain reaction-restriction fragment length polymorphism (PCR-RFLP) and ELISA, respectively.

**Results:**

Results of the current study showed a clear decline in circulatory VEGF-A levels in DFU subjects. VEGF-A was decreased in DFU subjects with the mutant “CC” genotype. The mutant “CC” of VEGF +405G/C was also found to be more susceptible to ulcer grade (III and IV) and major amputations.

**Conclusion:**

VEGF +405G/C SNP is associated with levels, infection severity, and amputation amongst South Indian DFU patients.

## 1. Introduction

The global spread of Type 2 diabetes mellitus (T2DM) and its late complications increases with a serious impact on public health as well as health care systems. It has been well estimated by the International Diabetes Federation (IDF) that, by the year 2017 about 425 million people were affected with T2DM and this figure is assessed to rise up to 628 million by the year 2045 [1]. Amongst various other T2DM complications, diabetic foot ulcer (DFU) is the most distressing one which frequently ends in nontraumatic foot amputation and with an increased mortality rate [2]. Indeed, earlier studies have well reported that the incidence of amputation among the diabetic population is 10 times higher than that of the nondiabetic population [3]. Although the etiology of a foot ulcer is multifactorial, one prime prognostic factor for the consequence of ulceration is the infection which may cause septic gangrene and thereby leads to foot amputation [4]. Thus, managing DFU entails a complete multidisciplinary method such as glycemia management, debridement, adequate antibiotic therapy, off-loading and surgical revascularization [5, 6]. Furthermore, when associated with various other T2DM complications, the genetics of DFU is not yet fully investigated. An earlier report from our laboratory on the South Indian race investigated the association of various single nucleotide polymorphisms (SNPs) in chemokines and cytokines genes with the severity and treatment outcomes of DFU [7]. Apart from the earlier report, there are hardly any other studies available to link SNPs in association with microbial infection load, ulcer severity, and treatment regimen for DFU. Indeed, another study by our team had already shed light on the genetic association of interleukin-6, tumor necrosis factor-*α,* and stromal-derived factor-1 SNPs in association with its circulatory cytokine levels among South Indian subjects with DFU [8].

Among several other growth factors that are intricated in the progression of late T2DM complications, VEGF has established mounting attention [9–11]. It is an effective proangiogenic growth factor that surges vascular permeability *in vivo* and triggers endothelial cells *in vitro* [12]. The gene encoding *VEGF* is situated on chromosome 6p21.3 and is highly polymorphic and has 8 exons and seven introns [13]. Earlier studies have well documented that VEGF is intricated in the pathogenesis of various T2DM complications [14, 15]. Studies had also indicated that the effect on SNPs has functional significance affecting the level of mRNA expression [16]. Further, circulatory levels were found elevated among T2DM and its late complications like DFU, diabetic retinopathy, and diabetic nephropathy [17–19]. To date, various variants have been identified in the *VEGF* gene, in which +405 G/C (rs2010963) SNP has a direct correlation with its circulatory levels. Despite many debatable reports discussing the association of *VEGF* gene +405 G/C (rs2010963) SNP and its circulatory levels, however, nil reports are seen to assess the association between the *VEGF* gene and its circulatory effect in subjects with DFU, particularly from our South Indian population.

Thus, the current study was designed to investigate the association of *VEGF* +405 G/C (rs2010963) in DFU subjects and correlate it with its circulatory levels, infection severity, and amputation rate.

## 2. Materials and Methods

### 2.1. Recruitment of Study Subjects

In this study, we registered a total of 754 patients that included 3 different groups, namely, Group I healthy controls (*n* = 297; M/F: 150/147), Group II consisting of T2DM patients (*n* = 242; M/F: 123/119), Group III consists of DFU patients (*n* = 215; M/F: 117/98). Further, Group III subjects were segregated into subgroups based on their grades (G1 (*n* = 84), G2(*n* = 60), G3(*n* = 45), and G4 (*n* = 26)), as per IDSA-IWGDF classification of foot infections [20]. All the study participants were unconnected participants of the South Indian race, after obtaining informed consent in agreement with the principles of the Declaration of Helsinki the study was initiated as per the institutional ethical clearance (Ref. No: IHEC/N-002/05/2020). The exclusion criteria were subjects with type 1 DM, gestational DM, autoimmune, and rheumatic disorders. Diagnosis of healthy controls and T2DM patients was well defined in our earlier laboratory publications and also from other reports [21–23]. Diagnosis of DFU was performed by a well-trained team of podiatrists who scored the gradings of infection as per the distinct criteria. Patients with DFU were investigated for the occurrence of osteomyelitis by the probe-to-bone test, microbiologic culture, and imaging procedures. 5 ml venous blood samples were collected in fasting condition to evaluate various clinical and genotypic analyses. Details on the anthropometric, demographic, and surgical procedures/interventions were recorded from the eligible participants. Further, the abbreviations for the acronyms used in the manuscript are detailed in Supplementary (S. Table 1).

### 2.2. Analysis of the Power of the Study

This study was undertaken with 75 subjects in each group. The sample size for this study was derived from the initial results with an estimated *P* < 0.05, with a power of 80% and a confidence interval (CI) of 95%.

### 2.3. Genotyping *VEGF*+405G/C Ser338Phe (rs2010963) SNP

The phenol-chloroform method was adopted for DNA extraction. PCR-RFLP was used to genotype the nonsynonymous SNP of *VEGF*+405G/C (rs2010963) [8]. The 304 bp segment containing SNP rs2010963 (p.Ser338Phe) in the VEGF gene was amplified by using the following primers; Forward: 5′-TGTGGATTTTGGAAACCAGCAGA-3″; Reverse: 5′-CGGTGTCTGTCTGTCTGTCCG-3″. As a next step, the amplicon was kept for restriction digestion using the BsmFI enzyme (New England Biolabs, MA). 193 and 111 bands were observed in the existence of a wild genotype (GG) and only one band of 304 bp was observed in the existence of a mutant genotype (CC). The heterozygous genotype (G/C) was confirmed in the presence of three bands 304, 193, and 111 bps. Plasma VEGF-A levels were quantitatively analyzed using Sandwich ELISA (R&D Systems, MN, USA) and expressed as pg/mL.

### 2.4. Statistical Analysis

SPSS-20 was used for all statistical purposes. Clinical data are represented as mean ± SD. Genotype distribution and allele frequency were compared across groups using an independence *χ*2 test with 2 × 2 contingency statistics [24]. The odds ratio (OR) was calculated with 95% CI as appropriate. The genetic association of genotypes with different stages of ulcer grade and the treatment regimens were carried out using backward logistic regression analysis. Initially, the odds ratios were calculated without including the confounding variables and then the significance of its association between the genotypes was tested by adding the confounding variables. *P* value <0.05 was considered to be statistically significant.

## 3. Results

### 3.1. Biochemical Investigations on the Recruited Participants

The details of the biochemical investigations were illustrated in Table 1. All the clinical investigations (except age) displayed a significant difference in T2DM patients when compared to healthy controls. On the other hand, fasting plasma glucose (FPG), postprandial plasma glucose (PPG), HbA1c, triglycerides (TGL), LDL-cholesterol (LDL-c), and urea were significantly elevated in DFU patients when compared to T2DM patients, whereas the other biochemical parameters did not show any statistical differences in the DFU subjects. With respect to other diabetic complications in DFU subjects, 37.2% had diabetic nephropathy, 20.9% had diabetic retinopathy, 16.7% had both retinopathy and nephropathy, 11.2% had myocardial ischemia, 8.4% had coronary artery disease, and 5.6% had cardiovascular disease.

Further, the DFU subjects were segregated as per their wound grades according to the well-established IDSA-IWGDF classification for foot infections. It was observed that there were 84 ulcers (39.1%) considered as Grade-I (uninfected), 60 ulcers considered Grade-II (mild; 27.9%), 45 ulcers considered Grade-III (moderate; 20.9%), and 26 ulcers considered Grade-IV (severe; 12.1%). Reports of microbiological samples showed infections caused by *Staphylococcus aureus* were 34.0%, *Pseudomonas aeruginosa* were 16.0%, *Klebsiella spp* were 13.0%, *Escherichia coli* were 11.0%, *Enterococcus faecalis* were 9.0%, *Proteus mirabilis* were 9.0%, and other infections were 8.0%, respectively.

### 3.2. Genetic Association of *VEGF* +405G/C Ser338Phe(rs2010963) with the Disease Phenotype

**Figure 1:**
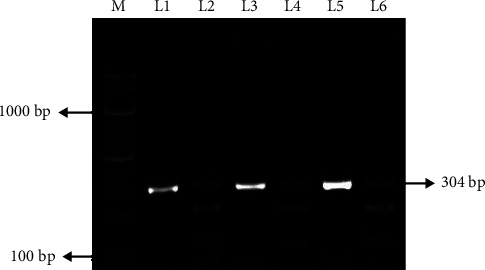
Agarose gel electrophoresis pattern of SNP in *VEGF* +405G/C (rs2010963) gene using PCR-RFLP PCR-RFLP results of *VEGF* +405G/C gene on 2.0% agarose gel. Marker (M): 100 bp molecular weight marker; lanes: L1, L3 and L5 indicate homozygous mutant with one band (304 bp PCR product); lanes: L2, L4, and L6 indicate heterozygote genotype with three bands (304 bp, 193 bp, and 111 bps).

A total of 754 subjects were genotyped for the *VEGF* +405G/C (rs2010963) SNP using the PCR-RFLP approach which is depicted in Figure 1. The bands for the GG genotype (wild) were visualized at 193 and 111 bps, the CC genotype (mutant) at 304 bp, and the GC genotype (hetero) at 304, 193, and 111 bps.

The allele and genotype frequencies of *VEGF* SNP were shown in Table 2. The genotypic distribution of these SNPs was found to be in Hardy–Weinberg equilibrium. The frequency of minor CC genotypes was increased in DFU subjects (14.88%), followed by T2DM (7.85%), compared to control participants (3.03%) (*P* < 0.0001). The frequency of the minor “C” allele showed significantly higher among DFU 24.88% and 17.36% in T2DM, respectively. Further, the DFU subjects were subdivided based on their ulcer grades and it was observed that the frequencies of the “C” allele of *VEGF* SNP were found to be significantly increased among Grade-IV infection (57.69%), followed by Grade-III subjects (32.22%) and Grade-II (22.50%), respectively, when compared to grade-I subjects (Table 3).

In addition, to reconfirm, the association of *VEGF* +405G/C in both T2DM and DFU patients was found by calculating an Odds Ratio (OR). In this logistic regression analysis, the “GG” genotype was taken as the reference genotype, and when the healthy control group was taken as a reference and compared with T2DM and DFU subjects, the results of the study showed that the “CC” genotype conferred significant risk towards DFU (OR: 6.0; 95% CI: 2.4–8.3; *P* = 0.0001) andT2DM (OR: 2.2; 95% CI: 1.1–5.2; *P* = 0.001) over the GG genotype, even after adjusting for its potential confounders like BMI, age, and gender. The unadjusted OR for the “C” allele in the DFU group was observed as 2.25 (95% CI: 1.63–3.1; *P* = 0.0001), for the T2DM group it showed 1.43 (95% CI: 1.0–2.0; *P* = 0.03), as compared to “C” allele of controls (Table 4). Next, the odds ratio was generated using the T2DM group as the reference type and compared it with DFU subjects. The study findings showed that the “CC” genotype confers significant susceptibility for DFU subjects with OR as 2.7 (95% CI: 1.0–3.5; *P* = 0.001) after adjusting for various confounding factors like BMI, age, and gender. The unadjusted OR for the “C” allele in DFU subjects was observed as 1.5 (95% CI: 1.1–2.1; *P* = 0.004), in comparison to “G” allele of T2DM subjects. As an overall result, the mutant “CC” genotype of *VEGF* +405G/C Ser338Phe (rs2010963) SNP exhibited a risk for both T2DM and DFU (Table 4).

### 3.3. Genetic Association of the *VEGF* +405G/C Ser338Phe (rs2010963) Genotypes with Ulcer Grade and Treatment Regimen in DFU

**Figure 2:**
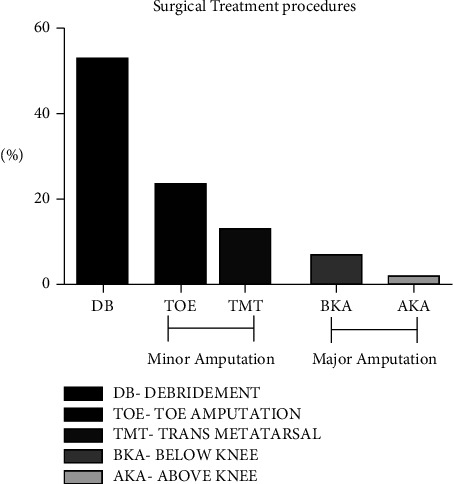
Surgical treatment procedures.

Table 5 shows the genetic association of the *VEGF* genotypes with the ulcer grades and treatment regimen in DFU subjects. Using backward logistic regression analysis, it was found that the recessive model genotype CC vs GG + GC conferred significant risk for Grades III and IV ulcer (OR: 4.3; 95% CI: 1.5–78.8; *P* = 0.0001) after adjusting for confounding variables like age, sex, and BMI.  Figure 2 shows the percentage of various surgical treatment regimens performed on the recruited. Out of a total of 215 DFU, debridement (DB) was the most common form of treatments-115 (53%) followed by minor amputation: toe amputation-52 (25%) and trans metatarsal amputation-28 (15%) and major amputation: below knee amputation-15 (7%) and above knee amputations-5 (2%). The genetic association of *VEGF* genotypes was computed with respect to the treatment procedures for DFU subjects using backward logistic regression analysis, it was found that subjects with recessive model genotype CC vs GG + GC conferred significant risk for major amputation procedures (OR: 1.9; 95% CI: 1.1–2.7; *P* = 0.004), after adjusting for various confounding factors (Table 5). Thus, our overall results showed that the mutant genotype “CC” was found to be more susceptible to ulcer grade (III and IV) and major amputations (below and above knee amputations).

### 3.4. Circulatory Plasma Levels of VEGF-A in the Study Participants

**Figure 3:**
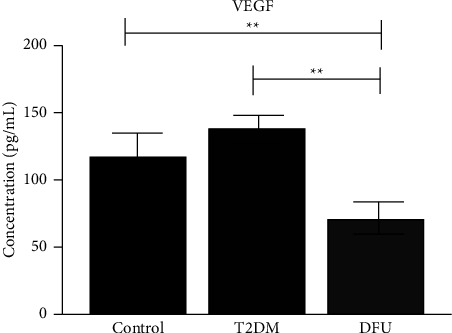
VEGF.

As can be seen in Figure 3 the median circulatory VEGF-A levels were found to be significantly decreased among DFU subjects (76.3 (56.3–81.8); *P* = 0.007), as compared to that of healthy group participants (118.6 (98.8–141.1)). Although the circulatory levels of VEGF-A were found to be increased in T2DM (135.6 (128.0–156.3); *P* = 0.006), we found a loss in statistical significance as in comparison with healthy controls. As a next step, the comparison was done between T2DM and DFU groups, and the results displayed a significant decrease in the levels of DFU (*P* = 0.004).

### 3.5. Association of *VEGF* +405G/C Ser338Phe (rs2010963) SNP with Circulatory Levels of Plasma VEGF-A

**Figure 4:**
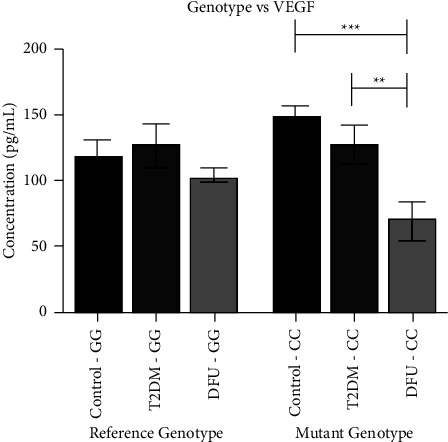
Genotype vs VEGF.

Figure 4 illustrates an association between *VEGF* +405G/C Ser338Phe (rs2010963) SNP and circulatory VEGF-A levels. To find this association, we segregated mean values of VEGF-A based on their genotype identified. In the case of the mutant “CC” genotype, our results explored the median VEGF-A levels to be significantly reduced in DFU subjects (60.2 (48.9–76.3), *P* = 0.003), in comparison with T2DM participants. On the other hand, the reference genotype “GG,” didn't show any significant difference in the median VEGF-A levels amongst both T2DM (128.0 (110.0–143.0), *P* = 0.48) and DFU (103.2 (99.4–108.8), *P* = 0.05), as compared with healthy controls participants (114.6 (107.8–135.3)).

## 4. Discussion

Earlier findings from our laboratory had reported an increased inflammatory cytokine that was associated with the circulation of DFU patients and thus impair the proper wound-healing process [25]. Thus, chronic inflammation which is coupled with infection delays wound healing and thereby results in amputations. On the other hand, VEGF effectively participates in vascular and angiogenic functions that are usually associated with the development of diabetic complications [26]. Hence, this present study explored the association of *VEGF* +405 G/C (rs2010963) in subjects with DFU which may provide a chance to measure the impact of a candidate gene on the progression of DFU and also, we have correlated the effect of SNP with its phenotypic impact on the levels, infection severity, and amputation.

Although numerous SNPs in the *VEGF* gene had been reported, the SNP at +405 G/C (rs2010963) in the 5′ untranslated region has a direct effect on the release of VEGF. Further, the GG genotype was also found to be associated with an elevated VEGF production among healthy persons [27]. To the best of our knowledge, the present study is the first one to explore the association of *VEGF* +405 G/C SNP genotype (rs2010963) and DFU along with infection severity and amputation rates. To date, there are hardly three case control studies available in the literature on *VEGF* SNP, particularly among DFU subjects. The first study by Amoli et al. [28] from Iranian DFU patients (but not with the same genotype chosen as per the present study), reported that the “C” allele of *VEGF* -278C/A was associated with the progression of DFU, however, one limitation of the study is that the VEGF levels in DFU patients were not measured and correlated with the clinical parameters of DFU patients. The second study by Li et al. [29] explored two different SNPS of *VEGF* (rs699947 andrs13207351) among Chinese Han DFU patients and reported that −2578 C/A (rs699947) conferred significant protection for the development of DFU. The apparent disparity between the aforementioned study and our study could be due to differences in race. The third study by Li et al. [17] reported that the GG genotype of *VEGF*-634G/C SNP conferred significant risk for DFU patients.

The results of the current study had shown that the CC genotype of *VEGF* +405 G/C SNP conferred significant risk for both T2DM and DFU patients. Our results are in agreement with a study that explored different disease conditions. Farhangi et al. [30] documented an association between the CC genotype of *VEGF* +405 G/C SNP and dietary diversity score among patients with metabolic syndrome. In contrast to the present study, Nikzamir et al. reported that the GG genotype of *VEGF* +405 G/C SNP is independently related to the progression of diabetic nephropathy [31]. Two controversial findings on different disease conditions in the same variant of *VEGF* +405G/C SNP from our similar race are available in the literature. Bhanoori et al., 2005 investigated that the +405G (but not +405C) allele had more chances of an increased risk for endometriosis among South Indian women [32]. Another study by Guruvaiah et al. [33] had also shown that the +405G/C in which the “G” allele conferred significant risk among South Indian polycystic ovary syndrome. Possible reasons for these discrepancies could be due to different disease subjects adopted for the study. Another reason for this disparity is the sample size in both the aforementioned studies was comparatively lesser than in the present study.

Another important aim is to estimate the plasma VEGF-A levels in DFU patients. The results showed that circulatory levels are significantly decreased among DFU subjects when compared to T2DM. This finding is in agreement with another study by Li et al., who had shown a decreased expression of VEGF levels in DFU patients when compared with the T2DM population from the Han Chinese population [17]. On the other hand, a study from a similar race to ours, but under a different disease condition had shown no association between *VEGF* +405 G/C SNP (rs2010963) and its levels among subjects with psoriasis [15].

Further, the results of the present study showed that the CC genotype of DFU patients was associated with a decrease in circulatory levels. This result was in accordance with an earlier study by Watson et al., who had conducted an *in vitro* study and identified a significant correlation between lipopolysaccharide-stimulated peripheral blood mononuclear cell VEGF protein production and *VEGF* +405 genotypes. It was also found that +405 CC showed decreased protein than +405 GG genotypes [34].

In addition, the present study was designed to observe the relationship between the *VEGF* +405 G/C SNP genotypes and correlate it with the outcome of treatment procedures for DFU. The present study results had shown that the mutant genotype “CC” was found to be more susceptible to develop higher DFU grades and amputations. Although reports on the association of *VEGF* (rs2010963) genotypes and treatment outcomes are less inconsistent, our results agree with an earlier study conducted by Viswanathan et al. on DFU from the South Indian race [7]. They showed that increased risk for developing higher grades of DFU and amputations among, IL-6 (-174G/C), SDF-1 (+801G/A), and TNF-*α* (-308G/A) genotypes. Thus, our present study on *VEGF* SNP has emerged as a strong risk factor for developing DFU.

## 5. Conclusion

To the best of our knowledge, this is the first study on *VEGF* +405 G/C SNP to steadily compare the genetic association with the outcome of ulcer severity and treatment procedures in an alarming disease condition such as DFU. However, the major limitation of our study is its cross-sectional nature. Going forward, our team will focus on assessing the physiological validity and relevance of the circulatory levels observed among the different grades of DFU. Therefore, the study concludes that VEGF +405 G/C SNP has a genetic susceptibility factor, which would assist in preventing amputation.

## Figures and Tables

**Table 1:** Clinical and biochemical characteristics of the study subjects.

Clinical parameters (*n* = 754)	Healthy controls (*n* = 297)	T2DM^Ϯ^ (*n* = 242)	DFU^ϮϮ^ (*n* *=* 215)
Gender (M/F)	150/147	123/119	117/98
Age (years)	51.1 ± 9.9	52.5 ± 11.1	50.8 ± 10.9
BMI (kg/m^2^)	23.7 ± 1.1	27.1 ± 4.5^*∗∗∗*^	27.4 ± 3.7
Systolic BP (mm Hg)	118.7 ± 12.1	129.3 ± 16.5^*∗∗∗*^	129.7 ± 18.6
Diastolic BP (mm Hg)	78.1 ± 8.5	87.7 ± 10.1^*∗∗∗*^	87.2 ± 9.9
Fasting plasma glucose (mg/dL)	101.8 ± 8.9	165.2 ± 56.4^*∗∗∗*^	171.3 ± 59.2^*∗∗∗*^
Postprandial plasma glucose (mg/dL)	121.7 ± 19.2	221.7 ± 57.4^*∗∗∗*^	234.2 ± 63.8^*∗∗∗*^
Glycated hemoglobin (%)	5.5 ± 0.4	9.1 ± 2.0^*∗∗∗*^	11.0 ± 2.1^*∗∗∗*^
Total serum cholesterol (mg/dL)	151.7 ± 23.9	178.3 ± 32.9^*∗∗∗*^	181.4 ± 33.5
Serum triglycerides (mg/dL)	118.5 ± 15.5	163.7 ± 49.1^*∗∗∗*^	170.5 ± 44.7^*∗∗*^
HDL-cholesterol (mg/dL)	42.3 ± 7.8	36.4 ± 8.1^*∗∗∗*^	35.5 ± 7.9
LDL-cholesterol (mg/dL)	85.9 ± 5.1	124.4 ± 27.5^*∗∗∗*^	132.5 ± 23.8^*∗∗∗*^
Urea (mg/dL)	20.5 ± 5.7	22.4 ± 5.1^*∗∗∗*^	30.5 ± 7.4^*∗∗∗*^
Creatinine (mg/dL)	0.8 ± 0.1	1.1 ± 0.1^*∗∗*^	1.1 ± 0.3

All data are reported as mean ± SD for continuous variables. BMI-Body mass index; SBP-systolic blood pressure; DBP-diastolic blood pressure; FPG-fasting plasma glucose; PPG-postprandial plasma glucose; HbA1c-glycated hemoglobin; HDL-high-density lipoprotein; LDL-low-density lipoprotein. ^*∗*^*P* < 0.05; ^*∗∗*^*P* < 0.01; ^*∗∗∗*^*P* < 0.001. Ϯindicates comparison was made between T2DM and healthy controls subjects. ^ϮϮ^indicates comparison was made between T2DM and DFU subjects.

**Table 2:** Distribution of genotype and allele frequencies of *VEGF* +405G/C (rs2010963) SNP in the study subjects.

Genotype/allele (*n* = 754)	Healthy controls (*n* = 297)	T2DM (*n* = 242)	DFU (*n* = 215)
GG	230 (77.44%)	177 (73.14%)	140 (65.12%)
GC	58 (19.53%)	46 (19.01%)	43 (20.0%)
CC	9 (3.03%)	19 (7.85%)	32 (14.88%)
G	518 (87.21%)	400 (82.64%)	323 (75.12%)
C	76 (12.79%)	84 (17.36%)	107 (24.88%)

Values are numbers (percentages).

**Table 3:** Distribution of genotype and allele frequencies of *VEGF* +405G/C SNP based on the severity of ulcer grades.

Genotype/allele	DFU (*n* = 215)				Chi-square test/fisher's exact test *P* value
	Uninfected/grade-I (*n* = 84)	Mild infection/grade-II (*n* = 60)	Moderate infection/grade-III (*n* = 45)	Severe infection/grade-IV (*n* = 26)	
GG	65 (77.38%)	40 (66.67%)	26 (57.78%)	9 (34.62%)	<0.0001
GC	17 (20.24%)	13 (21.67%)	9 (20.0%)	4 (15.38%)	
CC	2 (2.38%)	7 (11.67%)	10 (22.22%)	13 (50.00%)	
G	147 (87.50%)	93 (77.50%)	61 (67.78%)	22 (42.31%)	<0.0001
C	21 (12.50%)	27 (22.50%)	29 (32.22%)	30 (57.69%)	

**Table 4:** Genetic association of *VEGF* +405G/C (rs2010963) gene SNP in DFU subjects: odds ratio (OR) for minor alleles and their homozygous and heterozygous genotypes.

SNP (*n* = 745)	Healthy controls vs. T2DM		Healthy controls vs. DFU		T2DM vs. DFU	
	Unadjusted OR (95% CI)	Adjusted OR (95% CI)^a^	Unadjusted OR (95% CI)	Adjusted OR (95% CI)^a^	Unadjusted OR (95% CI)	Adjusted OR (95% CI)^a^
GG	Ref		Ref		Ref	
GC	0.96 (0.62–1.48), 0.87	0.84 (0.21–1.1), 0.79	1.03 (0.66–1.60), 0.89	0.89 (0.27–1.1), 0.82	1.06 (0.67–1.69), 0.78	0.7 (0.4–1.2), 0.001
CC	**2.72** ^*∗∗*^ (1.21–6.14), 0.01	**2.24** ^*∗∗∗*^ (1.14–5.27), 0.001	**5.59** ^*∗∗∗*^ (2.61–11.9), 0.0001	**6.02** ^*∗∗∗*^ (2.45–8.38), 0.0001	**2.05** ^*∗∗*^ (1.12–3.74), 0.018	**2.22** ^*∗∗∗*^ (1.09–3.51), 0.001
OR for minor “C” allele	**1.43** ^*∗∗*^ (1.02–2.0), 0.03		**2.25** ^*∗∗∗*^ (1.63–3.12), 0.0001		**1.57** ^*∗∗∗*^ (1.14–2.17), 0.005	

^**a**^Odds ratio (OR) adjusted for confounding factors (age, gender, and BMI). The figures in bold were significant. ^*∗∗*^*P* < 0.01; ^*∗∗∗*^*P* < 0.001.

**Table 5:** Genetic association of *VEGF* +405G/C (rs2010963) genotypes with ulcer grades and the outcome of treatment procedures for DFU.

SNP (*n* = 745)	Grades of ulcer		Outcome of treatment procedure	
	Grade I and II OR (95% CI)	Grade III and IV OR (95% CI)	Minor amputation OR (95% CI)	Minor amputation OR (95% CI)
GG vs GC + CC	Ref		Ref	
CC vs GG + GC	2.58 (1.9–42.7); 0.08	4.31 (1.5–78.8); 0.0001	1.24 (1.19–3.51); 0.83	1.97 (1.07–2.74); 0.004

## Data Availability

The data that support the findings of this study are available from the corresponding authors upon reasonable request.

## Additional Points

*Highlights of the Study* . (1) The mutant “CC” genotype of *VEGF* +405G/C Ser338Phe (rs2010963) SNP exhibited a significant risk towards T2DM and DFU. (2) VEGF-A levels were significantly decreased among DFU subjects when compared to T2DM. Further, VEGF-A was declined in DFU patients with mutant “CC” than the wild “GG” genotype. (3) The mutant genotype “CC” of *VEGF* +405G/C was found to be more susceptible to ulcer grade (III and IV) and major amputations (below and above knee amputations).

## Supplementary Materials

Supplementary Materials S.Table (1): List of abbreviations.
