# Supplementary material for: Vascular Endothelial Growth Factor Single Nucleotide Polymorphism +405 G/C (rs2010963) is associated with Levels, Infection Severity, and Amputation among South Indian Diabetic Foot Ulcer Patients
Source: Evid Based Complement Alternat Med. 2023 Apr 13;2023:2059426. doi: 10.1155/2023/2059426 (PMC10118891; doi:10.1155/2023/2059426)
Supplement: Supplementary Materials — S.Table (1): List of abbreviations. [file 2059426.f1.docx]

**S.Table (1): List of abbreviations**

| VEGF | Vascular Endothelial Growth Factor |
| --- | --- |
| SNP | Single Nucleotide Polymorphisms |
| DFU | Diabetic Foot Ulcer |
| T2DM | Type 2 Diabetes Mellitus |
| PCR | Polymerase Chain Reaction |
| RFLP | Restriction Fragment Length Polymorphism |
| IDF | International Diabetes Federation |
| IL-6 | Interleukin-6 |
| TNF-α | Tumour Necrosis Factor-α |
| SDF-1 | Stromal Derived Factor-1 |
| CI | Confidence Interval |
| OR | Odds Ratio |
| FPG | Fasting Plasma Glucose |
| PPG | Postprandial Plasma Glucose |
| HbA1c | Glycated Hemoglobin |
| TGL | Triglycerides |
| LDL-c | LDL-cholesterol |
| BMI | Body Mass Index |
| DB | Debridement |
| TA | Toe Amputation |
| TMA | Trans Metatarsal Amputation |
| BKA | Below Knee Amputation |
| AKA | Above Knee Amputation |
